# Cold atmospheric plasma treatment selectively targets head and neck squamous cell carcinoma cells

**DOI:** 10.3892/ijmm.2014.1849

**Published:** 2014-07-11

**Authors:** RAFAEL GUERRERO-PRESTON, TAKENORI OGAWA, MAMORU UEMURA, GARY SHUMULINSKY, BLANCA L. VALLE, FRANCESCA PIRINI, RAJANI RAVI, DAVID SIDRANSKY, MICHAEL KEIDAR, BARRY TRINK

**Affiliations:** 1 Department of Otolaryngology, Division of Head and Neck Cancer Research, School of Medicine, Johns Hopkins University, Baltimore, MD, USA; 2 Department of Obstetrics and Gynecology, University of Puerto Rico School of Medicine, Río Piedras, Puerto Rico; 3 Department of Mechanical and Aerospace Engineering, School of Engineering and Applied Science, The George Washington University, Washington, DC, USA

**Keywords:** head and neck squamous cell carcinoma, cold atmospheric plasma treatment, cold atmospheric plasma selectivity, head and neck squamous cell carcinoma adjuvant treatment, HPV-positive head and neck squamous cell carcinoma, HPV-negative head and neck squamous cell carcinoma

## Abstract

The treatment of locoregional recurrence (LRR) of head and neck squamous cell carcinoma (HNSCC) often requires a combination of surgery, radiation therapy and/or chemotherapy. Survival outcomes are poor and the treatment outcomes are morbid. Cold atmospheric plasma (CAP) is an ionized gas produced at room temperature under laboratory conditions. We have previously demonstrated that treatment with a CAP jet device selectively targets cancer cells using *in vitro* melanoma and *in vivo* bladder cancer models. In the present study, we wished to examine CAP selectivity in HNSCC *in vitro* models, and to explore its potential for use as a minimally invasive surgical approach that allows for specific cancer cell or tumor tissue ablation without affecting the surrounding healthy cells and tissues. Four HNSCC cell lines (JHU-022, JHU-028, JHU-029, SCC25) and 2 normal oral cavity epithelial cell lines (OKF6 and NOKsi) were subjected to cold plasma treatment for durations of 10, 30 and 45 sec, and a helium flow of 20 l/min^−1^ for 10 sec was used as a positive treatment control. We showed that cold plasma selectively diminished HNSCC cell viability in a dose-response manner, as evidenced by MTT assays; the viability of the OKF6 cells was not affected by the cold plasma. The results of colony formation assays also revealed a cell-specific response to cold plasma application. Western blot analysis did not provide evidence that the cleavage of PARP occurred following cold plasma treatment. In conclusion, our results suggest that cold plasma application selectively impairs HNSCC cell lines through non-apoptotic mechanisms, while having a minimal effect on normal oral cavity epithelial cell lines.

## Introduction

Head and neck squamous cell carcinoma (HNSCC) is the sixth most common type of cancer worldwide with an approximate 5-year survival rate of 50% (1). The prognosis for patients with HNSCC is determined by the stage of the tumor at presentation, as well as the presence of lymph-node metastases and distant metastases. Approximately one third of patients present with early-stage disease, whereas two thirds present with advanced cancer with lymph node metastases (2). Early-stage tumors are treated with surgery or radiotherapy and have a favorable prognosis. Thirty-five to 55% of patients with advanced-stage HNSCC remain disease-free 3 years after standard treatment (3). However, locoregional recurrence (LRR) develops in 30–40% of patients and distant metastases occur in 20–30% of HNSCC cases (4).

The standard of care for advanced tumors is surgery combined with adjuvant radiation therapy and/or chemotherapy. Survival outcomes are poor (40–50% five-year survival rates), and the treatment leads to morbidity (5). LRRs often requires a combination of surgery, radiation therapy, and/or chemotherapy, and metastatic disease is treated with chemotherapy. However, despite these therapeutic approaches, the control of LRR has been minimal. Therefore, addressing the underlying factors associated to locoregional disease will improve clinical management and decrease the burden of HNSCC.

Thermal and non-thermal plasma are ionized media that contain numerous active components, including electrons and ions, free radicals, reactive molecules and photons (6). Thermal plasma has been widely used to modify material surfaces; this modification is generally conducted in a vacuum (7,8). Cold atmospheric plasma (CAP), is a non-thermal plasma that has been shown to be highly effective in germicidal irradiation and sterilization, wound healing, blood coagulation, material surface modifications and crosslinking, as well as in the treatment of various diseases, including cancer (9–11).

In contrast to thermal plasma, CAP can reach high electron temperatures but very low gas temperatures associated with weak ionization rates (7). Thermodynamic equilibrium of electron self-collision in CAP occurs much faster than the equilibrium between electrons and larger particles, such as ions. Thus, the overall plasma temperature is much lower than the electron temperature, which is close to room temperature. Cold plasma has been used in biomedical research as it can reach ion temperatures closer to those at room temperature (12).

A number of studies have proposed the use of different cold plasma modalities for cancer treatment (10,13,14). Our laboratory recently examined the therapeutic potential of a manually-held CAP jet device in cancer cell lines and tumors, showing selective tumor eradication capabilities and apoptotic signaling pathway deregulation in melanoma cell lines and SCaBER-bearing mouse models (15). We demonstrated that CAP can potentially offer a minimally invasive surgical approach, allowing for specific cancer cell or tumor tissue removal without affecting the surrounding healthy cells and tissues, thus rendering it a promising technology for cancer therapy.

Despite the wide range of potential biomedical applications (16,17), the cell-specific effects of cold plasma treatment are not well understood at the molecular level (18). The generation of intracellular reactive oxygen species (ROS) leading to apoptosis has been proposed by different groups (19,20). Cellular necrosis (11) and senescence (21) have also been proposed to explain the mechanism of cold plasma treatment on cancer cells. Two possible underlying mechanisms for the high selectivity of CAP towards cancer cells can be attributed to the complex composition of CAP and the diverse characteristics of cancer and normal cells. While the specific mechanisms of action have not been identified, it is becoming apparent that cold plasma treatment may be more beneficial for some tumor sites than others.

The selective tumor eradication capabilities of the CAP jet device render it a potentially attractive adjuvant treatment for HPV-negative oropharyngeal squamous cell carcinoma patients who exhibit a higher rate of residual disease due to LRR when compared to HPV-positive patients (22). The aim of the present study was to examine whether CAP treatment for different exposure times shows selective tumor eradication capabilities in 4 HNSCC and 2 normal oral epithelial cell lines.

## Materials and methods

### Cell culture

The HNSCC cell lines (JHU-022, JHU-028, JHU-029, SCC25) were cultured in RPMI-1640 cell culture medium (Sigma, St. Louis, MO, USA) supplemented with 10% fetal bovine serum (Sigma) and Pen/Strep (100 units/ml penicillin and 100 μg/ml streptomycin) (both from Life Technologies, Grand Island, NY, USA). The 2 normal oral cavity epithelial cell lines (OKF6 and NOKsi) were grown in Keratinocyte-SFM (1X) supplemented with Keratinocytes Supplements (both from Gibco/Life Technologies). All cells were obtained from the Johns Hopkins University Head and Neck Cancer Division cell bank and incubated at 37°C in an atmosphere of 5% CO_2_.

### Cold plasma treatment

The CAP device, created in the School of Engineering and Applied Science of The George Washington University, contains 4 blocks. Block 1 is a DC power supply. Block 2 is a centrally powered electrode with a ground outer electrode wrapped around a quartz tube, which is part of the cold plasma production. Block 3 consists of a capacitor, a transistor and a timer; and block 4 is the helium gas supply, as previously described (6). Cold plasma treatments were carried out at 8 kV, using a helium flow of 10 l/min^−1^, with a distance of 3 cm from the plasma source to the cells, and treatment durations of 10, 30 and 45 sec.

**Figure 1:**
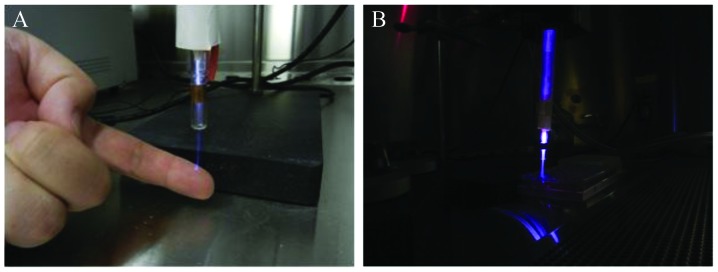
(A) Cold plasma is an ionized gas, in this case helium, produced at temperatures close to room temperature. The plasma jet is discontinuous and represents a series of propagating plasma bullets. (B) Treatments for this study were carried out at 8 kV, using a helium flow of 20 l/min^−1^ and a plasma source distance of 3 cm from exposed cells in 96-well plates.

We seeded the cells in 96-well plates and exposed them to cold plasma treatment for 10, 30 and 45 sec and a helium flow of 20 l/min^−1^ for 10 sec as a positive treatment control (Fig. 1). Following treatment, we transferred the cells to 2 sets of 6-well plates per cell line for MTT and clonogenic assays.

### MTT and clonogenic assays

MTT assay (Sigma) was performed on the plated cells 48 h after cold plasma application, according to the manufacturer’s instructions and the absorbance at 570 nm was measured. Clonogenic or colony formation assays were performed 7 days after treatment with cold plasma; colonies were visualized by staining with crystal violet (Sigma).

### Immunoblotting

Western blot analysis of PARP cleavage was performed 48 h after cold plasma treatment as follows: cell lysates were separated by SDS-PAGE on Tris-glycine gels and transferred to PVDF membranes (Bio-Rad Laboratories Inc., Hercules, CA, USA) The membranes were blocked with TBS-T + 5% non-fat dry milk and incubated overnight at 4°C with an antibody specific for PARP (Santa Cruz Biotechnology, Inc. Dallas, TX, USA). The membranes were washed and incubated with horseradish-peroxidase conjugated secondary antibodies. Protein detection was performed by enhanced chemiluminescence.

## Results

**Figure 2:**
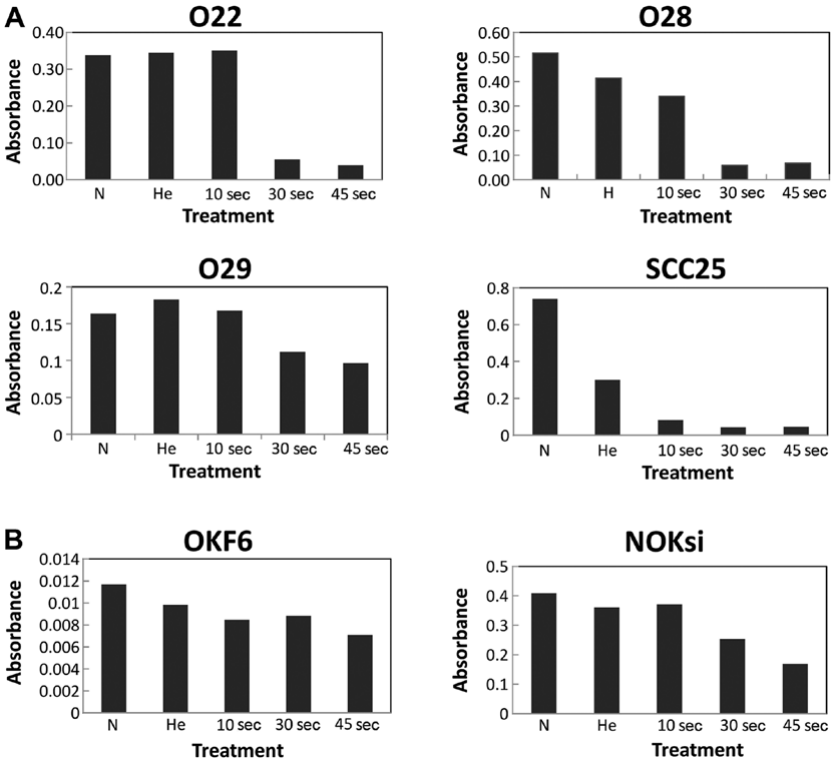
MTT assay results 48 h after cold plasma application. (A) Viability for head and neck squamous cell carcinoma (HNSCC) cell lines revealed that cold plasma selectively diminished the viability of SCC25 and JHU-O28 cells in a dose-response manner. (B) Viability results for normal oral cell lines. The normal oral cavity epithelial cell lines, OKF6 and NOKsi, were not affected by the cold plasma.

**Figure 3:**
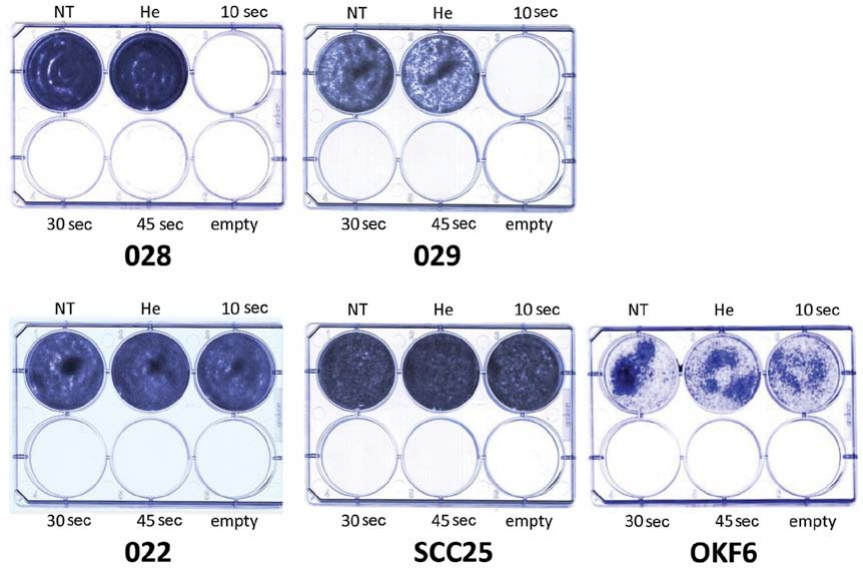
Representative colony formation assays for head and neck squamous cell carcinoma (HNSCC) cell lines (JHU-022, JHU-028, JHU-029 and SCC25) and normal oral cavity epithelial cell lines (OKF6).

**Figure 4:**
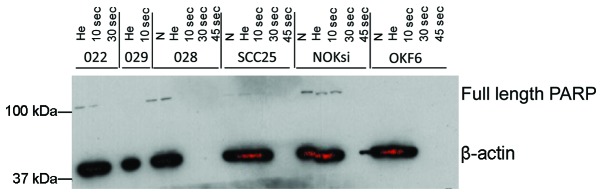
Western blot analysis 48 h after exposure to cold plasma. The blot does not show evidence that the cleavage of PARP occurred following cold plasma treatment.

The results from MTT assay revealed that cold plasma selectively diminished the viability of the SCC25 and JHU-O28 HNSCC cells in a dose- response manner (Fig. 2A). The JHU-O22 and JHU-O29 cells showed a diminished cell viability only after 30 and 45 sec of treatment. The viability of the OKF6 cells was not affected by the cold plasma, while the viability of the NOKsi cell lines was slightly diminished after 30 and 45 sec of treatment (Fig. 2B). The results of the colony formation assay also revealed a cell-specific response to cold plasma application. Exposure to helium flow for 10 sec did not impede colony formation. The JHU-O28 and JHU-O29 cells did not form any colonies following treatment with cold plasma for the 3 different time periods (Fig. 3). The JHU-O22, SCC25 and OKF6 cells only formed colonies following cold plasma treatment for 10 sec (Fig. 3). The NOKsi cells formed colonies following treatment with cold plasma at all 3 time periods (data not shown). Western blot analysis did not provide evidence that the cleavage of PARP occurred following cold plasma treatment (Fig. 4), suggesting that cold plasma application leads to selective cell death possibly through non-apoptotic pathways in HNSCC.

## Discussion

The main purpose of this study was to assess the selectivity of cold plasma in HNSCC cell lines and the mechanisms underlying this selectivity. Our results suggest that cold plasma application selectively impairs some HNSCC cell lines through non-apoptotic mechanisms, as the cleavage of PARP was not significantly altered in the treated cells, while having a minimal effect on normal oral cavity epithelial cell lines.

A number of studies have suggested possible molecular mechanisms for the effects of cold plasma on cancer cells. Several *in vitro* mechanisms have been suggested to be associated with a decrease in the expression of cell-surface proteins, such as integrins and FAK: cell detachment, the induction of apoptosis, the induction of senescence and the generation of ROS (17,20,21,23–25). The selective response of tumor cells to CAP may also be due to the phase of the cell cycle. It is known that the percentage of cancer cells is higher in the S phase of the cell cycle and this may render the cancer cells more susceptible to the effects of CAP, as previously shown in the 308 and PAM 212 cancer cell lines (26).

Mouse xenograft models of melanoma, bladder cancer, neuroblastoma and glioma treated with cold plasma have been found to have a decreased tumor volume and an increased survival rate (15,17,27). In addition, although some tumors recurred, their growth rate was reduced as compared to the tumors in the untreated mice.

In the present *in vitro* study, we observed that cold plasma application selectively targeted the HNSCC cell lines, JHU-O28 and SCC25, while it had a moderate effect on the JHU-022 and JHU-029 cells, and a minimal effect on normal oral cavity epithelial cell lines. The mechanisms appear to involve non-apoptotic pathways, as the cleavage of PARP was not detected following cold plasma treatment. One reason for the moderate effect on HNSCC JHU-022 and JHU-029 cells may be due to cold plasma-induced TP53 inactivation. In this regard, Skinner *et al* showed that disruptive *TP53* mutations render head and neck cancer cells more resistant to treatment with radiation (28). Since the mechanisms of action of cold plasma are not yet clearly known, it is tempting to speculate that cold plasma induced-*TP53* mutations may also cause resistance to treatment with cold plasma. However, our data suggest a mechanism of action independent of p53, as cold plasma had different effects on HNSCC regardless of the p53-status of these cells; the 3 JHU cell lines express wild-type p53 (29,30), while SCC25 cells express mutant p53 (31).

The control of LRR in HNSCC is of one of the most important clinical management goals. Failure to achieve this goal leads to complex clinical scenarios associated with persistent or recurrent disease at the primary tumor site or in regional lymph nodes. Furthermore, patients can develop metastatic disease, either as a consequence of the spreading from the primary tumor before the initial diagnosis or from treatment-resistant persistent/recurrent locoregional disease. Both of these clinical scenarios (persistent/recurrent locoregional or metastatic disease) represent extremely difficult management problems (32). Salvage treatment is possible but frequently unsuccessful, particularly in patients in whom macroscopic disease is evident at or within 6 months after the end of initial chemoradiotherapy. Salvage treatment usually entails both acute and long-term morbidity (33). Systemic metastatic disease may be palliated by cytotoxic chemotherapy, biological agents or low-dose radiotherapy, but remains incurable with a median survival of approximately 6–9 months (34). Other therapies include simultaneous chemoradiotherapy, and the combination of radiotherapy and targeted therapies (e.g., EGFR antibody, cetuximab) (35,36). However, despite these therapeutic approaches, locoregional control and survival rates have shown only a modest increase.

Patient mortality with HPV-negative HNSCC is primarily driven by tumor cell radioresistance leading to LRR. Overall and disease-specific survival is higher in patients with HPV-positive HNSCC tumors (37), which, as a distinct molecular and pathologic subtype, displays an average of 4 somatic mutations per tumor, while HPV-negative HNSCC tumors harbor 20. HVP-positive HNSCC patients have a different molecular profile than HPV-negative patients, which may modulate their sensitivity to cold plasma. For example, HPV-positive HNSCC patients usually do not have *TP53* mutations in their tumors, but the cell cycle is still deregulated in these patients, as the E6 HPV protein silences TP53 (38,39). *CDKN2A*, a principal cyclin-dependent kinase inhibitor that decelerates the cell cycle, is lost in HPV-negative HNSCC (40) and amplified in HPV-positive HNSCC (41). All the HNSCC cell lines we used in this study were HPV-negative. Thus, cold plasma may be successfully used as an adjuvant treatment for HPV-negative HNSCC patients with or without p53 mutations.

In a recent study by Wang *et al* (6), it was shown that, due to the complex composition and parameters of CAP, the high selectivity towards cancer cells may vary. In fact, the various components that compose the CAP as a variety of ROS, reactive nitrogen species, charge particles and UV, and parameters, such as voltage, resistance, plasma emission and power may alter the response of cells to treatment, thus promoting specific chemical reactions between charged particles and living cells, triggering intracellular biochemical reactions. In that study by Wang *et al,* the CAP treatment parameters were optimized to selectively kill human metastatic breast cancer (BrCa) cells, while minimally affecting healthy human bone marrow mesenchymal stem cells (MSCs) (6). Similarly, in the present study, we showed that it is feasible to fine-tune the settings of CAP to specifically target cancer cells, while leaving the adjacent normal tissue unharmed.

Cold plasma treatment represents an alternative means to selectively targeting HNSCC cells, while having a minimal effect on the normal adjacent tissue and a feasible therapeutic strategy if coupled with endoscopic technology. It could also be potentially used in the first-line eradication of small malignant growths and as an adjuvant irradiation treatment of malignant tissue prior to surgery and surgical margins after surgery. Cold plasma represents an alternative adjuvant therapy that may lead to a reduction in LRR, particularly in HPV-negative patients and, as such, it warrants further investigation.
